# Evidence of increasing Leu-Phe knockdown resistance mutation in *Anopheles gambiae *from Niger following a nationwide long-lasting insecticide-treated nets implementation

**DOI:** 10.1186/1475-2875-7-189

**Published:** 2008-09-25

**Authors:** Cyrille Czeher, Rabiou Labbo, Ibrahim Arzika, Jean-Bernard Duchemin

**Affiliations:** 1 Parasitology Unit, CERMES (Centre de Recherche Médicale et Sanitaire), Institut Pasteur International Network, Niamey, Niger

## Abstract

**Background:**

At the end of 2005, a nationwide long-lasting insecticide-treated net (LLIN) distribution targeting the most vulnerable populations was implemented throughout Niger. A large number of studies in Africa have reported the existence of anopheline populations resistant to various insecticides, partly due to knockdown resistance (*kdr*) mutations, but few operational wide-scale control programmes were coupled with the monitoring of such mutations. The distribution of the *kdr-west *(*kdr-w) *Leu-Phe mutation was studied in *Anopheles gambiae *s.l. populations from Niger and temporal variations were monitored following the nationwide LLIN implementation.

**Methods:**

Mosquitoes were collected from 14 localities during the wet seasons of 2005, 2006 and 2007 with additional sampling in the capital city, Niamey. After morphological identification of *Anopheles gambiae *s.l. specimens, DNA extracts were used for the determination of species and molecular forms of the *Anopheles gambiae *complex and for the detection of the *kdr-w *mutation.

**Results:**

Around 1,500 specimens collected in the three consecutive years were analysed. All *Anopheles arabiensis *specimens analysed were homozygous susceptible, whereas the few *Anopheles gambiae *S forms exhibited a high overall *kdr-w *frequency. The M form samples exhibited a low overall *kdr-w *frequency before the LLIN distribution, that increased significantly in the two wet season collections following the LLIN distribution. Higher *kdr *frequencies were repeatedly noticed within host-seeking females compared to resting ones in indoor collections. In addition, preliminary results in M form urban populations from Niamey showed far higher *kdr *frequencies than in all of the rural sites studied.

**Discussion:**

This study describes the first case of *kdr *mutation in *Anopheles gambiae *populations from Niger. It is suspected that the LLIN have caused the important temporal increase of *kdr-w *mutation observed during this study. While the *kdr *mutation is still found at a low level, this rapid increase could potentially lead to high *kdr *frequencies within a few years.

**Conclusion:**

These results are of prime importance in the effort to document multiple effects of operational control programmes on mosquito vectors, and to conceive sustainable control strategies for future malaria control programmes.

## Background

*Plasmodium falciparum*, one of the deadliest pathogens circulating in Niger and many other sub-Saharan countries, is contributing significantly to the high estimated child mortality rate (United Nations Children's Fund 2005). The major vectors responsible for *P. falciparum *transmission in Niger are *Anopheles gambiae *sensu lato (s.l.) and *Anopheles funestus*, the former being far more widespread over the country whereas the last is found in more limited areas [1]. Of the sibling species and forms constituting the *Anopheles gambiae *complex, *Anopheles arabiensis *and both molecular M and S forms of *An. gambiae *sensu stricto (s.s.) have been found to date in Niger [2,3].

At the end of 2005, a nationwide long-lasting insecticide-treated net (LLIN) distribution targeting the most vulnerable members of the population was organized in conjunction with an integrated poliomyelitis vaccination campaign [4]. This LLIN coverage aimed to reduce morbidity and mortality caused by malaria clinical cases. Such a reduction can be observed when the bed net usage rate in the population is high enough [5-9]. Although bed nets were already quite commonly used by the population, principally for protection against biting nuisance mosquitoes, this campaign strongly increased insecticide-treated bed net usage in households all over the country [10].

The effectiveness of bed nets impregnated with pyrethroid insecticides derives from several mechanisms that decrease the probability of a host-seeking female mosquito to succeed in taking a blood-meal. Besides the physical barrier constituted by the net, a deterrent effect limits unfed mosquitoes entering houses where a treated net is present, and in case of tarsal contact with the treated net, the insecticide compound could repel, hurt, or kill the mosquito. These properties have led to substantial reductions in indoor mosquito densities, and biting, feeding and survival rates in field trials [11-14] and experimental huts evaluations [15-18]. Some of these studies have also demonstrated a reduction in *P. falciparum *transmission [11,13,14].

However, a number of studies throughout Africa have reported the existence of anopheline populations resistant to various insecticides [15,19-23] involving two major types of resistance mechanisms : insecticide target-site insensitivity due to single nucleotide polymorphisms (SNP), and increased enzymatic metabolization of insecticidal compounds [24]. Two SNP at amino-acid position 1014 in the voltage-gated sodium channel gene have been described in *An. gambiae *[25,26], leading to amino-acid substitutions involved in reducing the affinity of DDT and pyrethroid insecticides for their target site in the insect nervous system. These SNP, named *knock-down resistance *(*kdr*) mutations in relation to their phenotypic expression, were found in many field populations of *An. gambiae *s.s. and *An. arabiensis *covering West, Central and East Africa [27-50]. In West and West-Central Africa, it was shown that the *kdr-w *(or L1014F) allele was far more frequent and widespread in the S molecular form of *An. gambiae *[23,27-29,32-38,50], whereas only few M form populations from a limited area near the Gulf of Guinea presented *kdr-w *alleles at low frequencies [23,31,32,36-38,50], except in few urban and peri-urban coastal areas where it reached high frequencies [20,35,40-45]. However, to date there have been almost no studies on *kdr *mutations in sahelian *An. gambiae *populations except one in Burkina Faso that included two sites in the sahelian zone of the country [32]. No *kdr *M forms were found whereas one village exibited *kdr-w *S forms.

The monitoring of insecticide resistance in malaria vectors is of prime importance especially where control programmes are planned or already running, in order to assess potential selection effects of insecticidal compounds on vector populations, and to take appropriate measures such as switching to other classes of compounds. For this goal, the presence and frequency of the *kdr *mutations constitute a valuable and useful resistance marker for two main reasons. First, it provides an early warning of resistance development as the mutation arises well before any effect on phenotype can be detected in a population [51]. Indeed, the expression of the 24 h-survival diagnostic phenotype [52] appear to be recessive [45,53]. Therefore, a population presenting a low *kdr *frequency mainly in heterozygous state is likely to show a high mortality rate during bioassays. As further evidences supporting the advantage of *kdr *genotyping over bioassays for emergent resistance detection, Chandre *et al*. [53] found a significant mortality reduction only when heterozygous females proportion reached 60%, and a significant increase of Knockdown time (KdT) only with 40% heterozygous females. Secondly, the *kdr *mutations seem to be well correlated with resistance phenotype [25,38,45,53] in both *An. gambiae *molecular forms, even if metabolic resistance mechanisms could also be involved in increased tolerance to pyrethroids [27].

Several authors have studied the effect of insecticide treated nets (ITNs) with pyrethroids on *An. gambiae *populations and the possible selection of *kdr *alleles either in experimental huts trials [18,45,53,54] or laboratory experiments [45,53], helping in the understanding of advantages conferred by the mutations on survival and blood-feeding in areas of ITN use. However, few data are available on long-term and/or large-scale ITN coverage effect on resistance and *kdr *mutations in natural settings. In East Africa, no selection effect of long-term ITNs use on phenotypic resistance was noticed [55,56], whereas Stump *et al*. [57] reported a significant increase of *kdr-east *mutation frequency in *An. gambiae *S form populations from Kenya after four years of ITNs community use. In West-Central Africa, a rapid increase of *kdr-w *mutation was observed in M forms from the island of Bioko following a large-scale insecticide residual spraying (IRS) programme [41,58].

The distribution of the *kdr-w *mutation was studied in *An. gambiae *s.l. populations from the sahelian area of Niger during three consecutive wet seasons, allowing to monitor temporal variations following the nationwide LLIN coverage implemented after the first collection event. This study reports the first documented case of *kdr *mutation in *An. gambiae *populations of Niger and provides crucial information about potential effects of wide-scale LLIN coverage on *kdr *mutation selection.

## Methods

### Study area

Niger is a West-African country spanning three bioclimatic zones, with a marked aridity gradient from the southern sahelo-sudanian zone (> 500 mm rainfall per year) to the northern pre-Saharan zone (< 250 mm rainfall per year). Located between these zones, the sahelian zone represents the northern limit of malaria endemicity in the region, and has relatively high human population densities compared to the north of the country, where malaria is more marginal and epidemic-prone and where *Anopheles *species population densities are very low, highly variable and heterogenous (in prep.).

Apart from describing the distribution of the *kdr-w *mutation in *An. gambiae *s.l. populations in Niger, we monitored its potential variations in frequency and spatial distribution following the countrywide LLIN coverage implemented by the end of 2005 that targeted children under five years of age. The nationwide extent of the campaign prevented us from monitoring control sites without LLIN usage, and the only available methodology was to study the temporal evolution of the *kdr-w *mutation in selected sentinel sites before and after the campaign.

### Study design and mosquito collections

**Figure 1:**
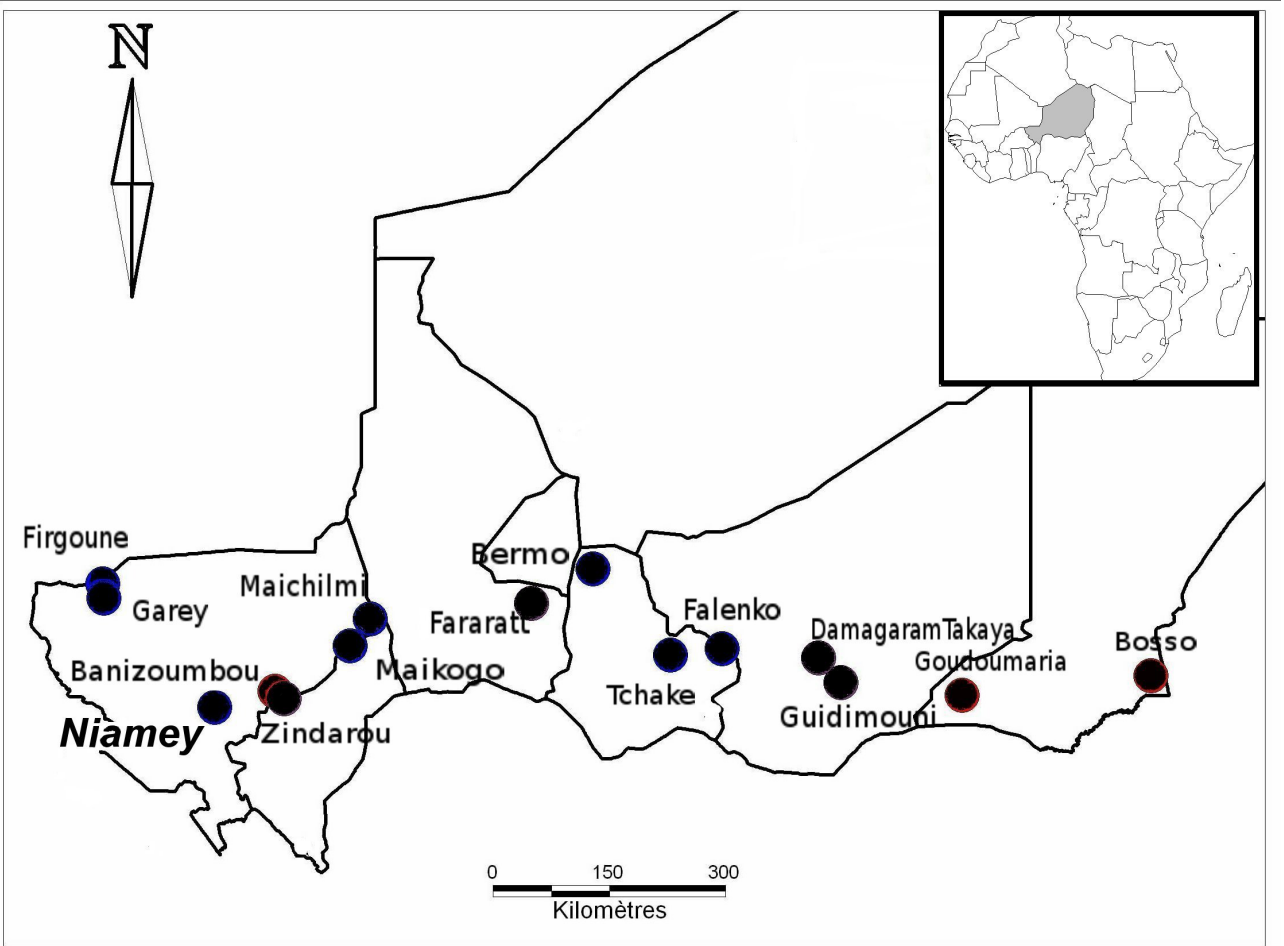
Map of the study area and collection sites.

Mosquitoes were collected from 14 localities situated in the sahelian zone during the wet season (between July and September) of 2005, 2006 and 2007 (Figure 1). The 2007 collections were made in eight of the 14 initial study sites because of logistical constraints. This sampling scheme allowed us to collect specimens from West to East over a distance of around 1,700 km. In each village, landing catches on human adult male volunteers and indoor spray catches with pyrethroids (Mobil insecticide, Mobil Oil Nigeria, Lagos, Nigeria) were employed following standardized procedures and with agreement of the National Ethics Committee. The 2005 collections were made before the implementation of the nationwide LLIN coverage to provide baseline *kdr *mutation frequency data, whereas the 2006 and 2007 collections were made approximately 7–8 months and 19–20 months after the LLIN distribution, respectively. Additionally, some larval samples collected in Niamey (the capital city) during the 2003 and 2007 wet seasons were included in the study.

### Laboratory processing and data analysis

Mosquito species were morphologically identified in the field and put in 96-well microplates with dessicant, and stored between -20 to -28°C in the laboratory before processing. Some specimens identified as *An. gambiae *s.l. were processed for DNA extraction from four to six legs using Chelex-100 resin (Sigma-Aldrich, St. Louis, MO, USA), while whole bodies were kept for transmission studies. DNA extracts were used for the determination of species and molecular forms of the *An. gambiae *complex by Polymerase Chain Reaction – Restriction Fragment Length Polymorphism (PCR-RFLP) assay [59], allowing discrimination of *An. arabiensis*, *An. gambiae *S form, and *An. gambiae *M form. These DNA extracts were then subjected to PCR assay for detection of the *kdr-w *mutation [25] with some modifications. As already done [49] and to get a more reliable genotype determination, we performed two different PCRs, one with primers Agd1 and Agd3 for the detection of the mutant allele, and the other one with primers Agd2 and Agd4 to detect the wild-type allele. Genotypes of every specimen harbouring at least one *kdr-w *allele were confirmed in a second run of both PCR, and a third run was done if the two first results were discordant. The *kdr-east *(Leu-Ser) mutation was not searched for as it is very rare in *An. gambiae *M forms [15,37,38,50]. The conformance of genotypic frequencies with Hardy-Weinberg equilibrium expectations was determined by exact test [60] as computed in Arlequin version 3.1.1 [61]. All other statistical analysis were performed online with OpenEpi version 2.2.1 [62]. One-tail p-values were employed for Chi square and Fisher exact tests considering the hypothesis of *kdr *frequency increase over the study period. Also, it should be noted that allelic frequency of the *kdr-w *mutation (number of chromosomes presenting the mutation divided by total number of chromosomes analysed) is always presented rather than frequency of *kdr *specimens (presenting one or two copies of the mutation).

## Results

### Global results

One thousand five hundred and seventeen specimens collected in the three consecutive years and different sites were analysed for the species/forms and the *kdr-w *mutation. 54 individuals (3.6%) could not be genotyped for the *kdr-w *mutation after two to three attempts, and were excluded from further analysis. *An. arabiensis *and *An. gambiae *M form were found in sympatry in all 14 collection sites and all years except in the village of Bosso where only *An. arabiensis *was found. Also, very few *An. gambiae *M forms were repeatedly collected in Guidimouni. Consequently, detailed *kdr *results for *An. gambiae *M forms will be presented for 12 villages and Niamey. However, important variations of abundances and relative proportions of these two species were observed throughout the country (in prep.), leading to the analysis of small sample sizes in some locations or years for either one or both species. The reliability of the method employed to distinguish *kdr-w *genotypes was good, with 83% of concordance between first and second PCR runs considering only *kdr *M forms from rural areas (n = 45). In addition, on 26 *kdr *M forms from Niamey (12 homozygotes and 13 heterozygotes), all gave concordant results between the two runs except one (*kdr/kds *in the first assay, *kdr/kdr *in the second one) giving 96% of concordance.

All 456 *An. arabiensis *specimens analysed were homozygous susceptible *kds/kds *(n = 186 in 2005; n = 139 in 2006; n = 131 in 2007).

Only 19 *An. gambiae *S form specimens were found clustered in four sites (Garey, Banizoumbou, Zindarou and Fararatt), exhibiting an overall *kdr-w *frequency of 50.0%, with five homozygous *kdr/kdr *genotypes, nine heterozygous *kdr/kds *genotypes, and five homozygous *kds/kds *genotypes. The proportion of these genotypes conformed to Hardy-Weinberg equilibrium expectations. Only this global result is given because the low sample size precluded any spatial or temporal variation analysis.

### Dynamics of *kdr *frequency in *An. gambiae *M forms

Of the 986 *An. gambiae *M form specimens analysed, 42 specimens harboured one copy of the *kdr-w *mutation, whereas only three specimens were homozygous for the mutant allele, giving an overall *kdr-w *frequency of 2.4%. However, the mutation was not evenly distributed in space and time, and the proportion of sites where at least one *kdr*-harbouring mosquito was found within sufficient sample sizes varied from 5/11 sites in 2005 (45.5%) to 6/8 sites in 2006 (75%), and reached 6/6 sites in 2007 (100%). The genotype distribution was in accordance with Hardy-Weinberg proportions for each year's collection as a whole (all sites).

In the collection of 2005, corresponding to the pre-LLIN period, the mean *kdr-w *mutation frequency was 0.5% (n = 537) (Table 1). In each of the five sites where the mutation was detected, only one heterozygous *kdr-w *carrier was found, giving frequency estimates ranging from 0.6% (n = 86) to 1.6% (n = 32). The higher *kdr-w *frequency (8.3%) found in Damagaram Takaya was calculated for six individuals, and may not be representative of the population due to the small sample size.

**Table 1:** kdr-w allelic frequency in An. gambiae M forms by site and year.

	2005		2006		2007		overall	
Sites	n	kdr freq (%)	n	kdr freq (%)	n	kdr freq (%)	n	kdr freq (%)
		[95% CI]		[95% CI]		[95% CI]		[95% CI]
Firgoune	47	0.00	17	0.00	29	10.34	93	3.23
		[0.0–3.8]		[0.0–10.3]		[3.9–21.2]		[1.2–6.9]
Garey	54	0.00	35	0.00	-		89	0.00
		[0.0–3.4]		[0.0–5.1]				[0.0–2.1]
Banizoumbou	55	0.00	52	2.88	26	7.69	133	2.63
		[0.0–3.3]		[0.6–8.2]		[2.1–18.5]		[1.1–5.3]
Zindarou	49	1.02	71	0.70	65	4.62	185	2.16
		[0.0–5.6]		[0.0–3.9]		[1.7–9.8]		[0.9–4.2]
Maichilmi	77	0.00	10	5.00	-		87	0.57
		[0.0–2.4]		[0.1–24.9]				[0.0–3.2]
Maikogo	86	0.58	19	7.89	34	8.82	139	3.60
		[0.0–3.2]		[1.7–21.4]		[3.3–18.2]		[1.7–6.5]
Fararatt	71	0.70	53	0.94	19	7.89	143	1.75
		[0.0–3.9]		[0.0–5.1]		[1.7–21.4]		[0.6–4.0]
Bermo	42	0.00	4	0.00	-		46	0.00
		[0.0–4.3]		[0.0–36.9]				[0.0–3.9]
Tchake	32	1.56	3	0.00	-		35	1.43
		[0.0–8.4]		[0.0–45.9]				[0.0–7.7]
Falenko	13	0.00	-		-		13	0.00
		[0.0–13.2]						[0.0–13.2]
Damagaram	6	8.33	5	10.00	-		11	9.09
		[0.2–38.5]		[0.3–44.5]				[1.1–29.2]
Guidimouni	3	0.00	1	50.00	4	87.50	8	50.00
		[0.0–15.9]		[1.3–98.7]		[47.3–99.7]		[24.6–75.3]

Total	537	0.47	272	2.02	177	9.04	986	2.43
		[0.2–1.1]		[1.0–3.6]		[6.3–12.5]		[1.8–3.2]

Six out of 12 villages were analysed in 2007. The study sites are presented following their longitudinal position, from West to East. Two sites (Goudoumaria and Bosso) where too few An. gambiae s.s. were found are not shown. n: specimens sample size. Kdr freq: kdr-w allelic frequency (percentage). 95% CI: 95% Confidence Interval

The mean *kdr-w *mutation frequency significantly increased (X^2 ^= 8.935, p = 0.0014, df = 1) in the 2006 collection (eight months after the LLIN distribution), as 2.0% of all M form specimens tested (n = 272) (Table 1) harboured the *kdr-w *allele in the heterozygous state only. Within the six locations where *kdr*-*w *carriers were found, the frequency of the mutation varied from 0.7% (n = 71) to 7.9% (n = 19). The site that had shown the highest *kdr *frequency within six individuals in 2005 gave similar results in 2006 (n = 5), indicating that the observed *kdr *frequency within only a few individuals might correctly estimate its real occurence in that local population. Also, the 50.0% *kdr *frequency found in Guidimouni must be interpreted with caution as the M form sample represents only one heterozygous female.

In the 2007 wet season, the mean *kdr *frequency increased again in M form populations reaching 9.0% (X^2 ^= 23.16, p << 0.001, df = 1) (Table 1), and was found in the resistant homozygous state for the first time, in the village of Guidimouni. The number of M form specimens was again low in this locality (n = 4), and all were *kdr *carriers with three homozygous *kdr/kdr *females, giving the highest *kdr *frequency found during this study (87.5%). All five other sites presented *kdr *carriers, with allelic frequencies ranging from 4.6% (n = 65) to 10.3% (n = 29).

Eight of the fourteen initial villages where sampled in 2007. Five of these presented enough sufficient M form sample sizes. The global temporal *kdr-w *frequency variation was also studied considering only those five sites. The results were quite similar, and with all five sites taken together, a highly significant increase of *kdr-w *frequency was detected (X^2 ^= 40.98, p << 0.001, df = 2), from 0.5% in 2005 to 1.9% in 2006 and 7.2% in 2007. Considering each site individually, only Maikogo presented a significant *kdr-w *frequency increase in the first wet season after the campaign (Fisher exact test, p = 0.019). In the second wet season after the campaign, *kdr *frequency increased significantly in Zindarou (Fisher exact test, p = 0.047) whereas significance was almost reached in Fararatt and Firgoune (Fisher exact test, p = 0.056 and 0.057, respectively). When comparing 2005 *versus *2007 collections, all five sites except Zindarou presented a significant increase of *kdr *allelic frequency.

### *Kdr *frequency analysis by collection methods

**Figure 2:**
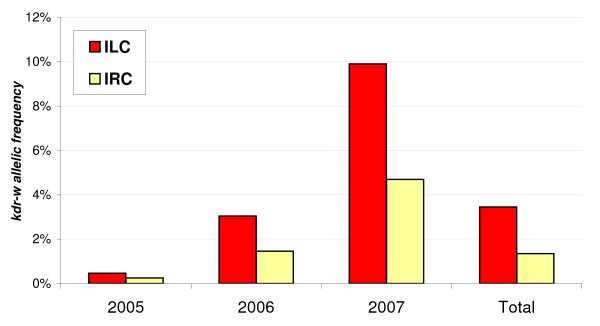
***kdr-w *allelic frequency in *An. gambiae *M forms by year and method of collection**. Averaged *kdr *frequency over all rural sites (13 villages in 2005 and 2006, 6 villages in 2007). ILC: Indoor Landing Collections. IRC: Indoor Resting Collections

The above results were presented without differentiation of collection methods, however they are meaningful because the proportion of tested individuals collected by each method was homogenous for the different years of collection (X^2 ^= 1.439, p = 0.487, df = 2 for five sites from Table 2; X^2 ^= 5.377, p = 0.0679, df = 2 for all sites). However, when analysing separately sub-samples of M forms collected by different methods, we found different *kdr-w *frequencies, with a trend towards higher values for host-seeking mosquitoes collected by landing catches inside dwellings compared to indoor resting mosquitoes collected by spray catches (Figure 2). This trend was seen for each year but the difference was statistically significant only for 2007 (X^2 ^= 3.232, p = 0.036, df = 1) and for global results over all years (X^2 ^= 7.392, p = 0.003, df = 1). In addition, we observed quite constantly around two-fold higher *kdr-w *frequencies in host-seeking females.

**Table 2:** kdr-w allelic frequency in An. gambiae M forms by site, year and method of collection.

		2005		2006		2007		overall	
Sites	Collection	n	freq kdr (%)	n	freq kdr (%)	n	freq kdr (%)	n	freq kdr (%)
Firgoune	ILC	24	0.00	5	0.00	21	14.29	50	6.00
	IRC	23	0.00	8	0.00	8	0.00	39	0.00
	total indoor	47	0.00	13	0.00	29	10.34	89	3.37
Banizoumbou	ILC	12	0.00	18	5.56	-		30	3.33
	IRC	35	0.00	24	2.08	26	7.69	85	2.94
	total indoor	47	0.00	42	3.57	26	7.69	115	3.04
Zindarou	ILC	22	0.00	52	0.96	50	5.00	124	2.42
	IRC	6	0.00	2	0.00	15	3.33	23	2.17
	total indoor	28	0.00	54	0.93	65	4.62	147	2.38
Maikogo	ILC	45	1.11	8	12.50	27	11.11	80	5.63
	IRC	15	0.00	7	7.14	7	0.00	29	1.72
	total indoor	60	0.83	15	10.00	34	8.82	109	4.59
Fararatt	ILC	35	0.00	15	0.00	7	14.29	57	1.75
	IRC	32	0.00	31	1.61	10	5.00	73	1.37
	total indoor	67	0.00	46	1.09	17	8.82	130	1.54

total	ILC	140	0.36	99	3.03	105	9.05	344	3.67
	IRC	111	0.00	72	2.08	40	2.50	223	1.81
	total indoor	251	0.20	171	2.63	145	7.24	567	2.88

The table presents data from the five sites analysed for the three years and with sufficient sample size. n: sample size. freq kdr: kdr-w allelic frequency (percentage). ILC: Indoor Landing Collections. IRC: Indoor Resting Collections

By focusing again on the five villages used for temporal variation analysis, but considering only host-seeking females collected inside, the global increase of *kdr-w *frequency from 2005 to 2007 was also highly significant (X^2 ^= 25.35, p << 0.001, df = 2) (Table 2), however it was not the case when considering only resting females, giving only an increasing trend between 2005 and 2006 (Fisher exact test, p = 0.060).

### Preliminary results for urban M form populations

**Figure 3:**
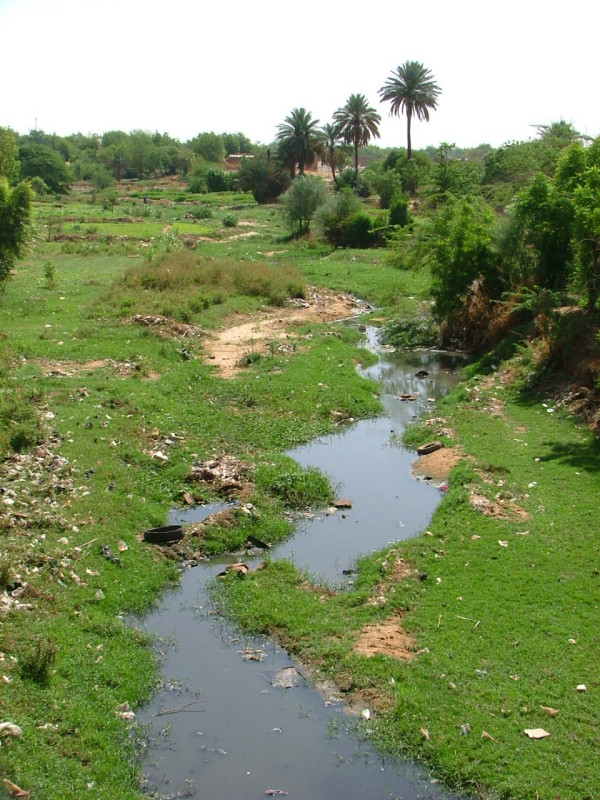
**View of the Gountou Yena stream**. Picture taken in Niamey during early wet season (june 2008), showing the small stream surrounded by small-scale gardening areas (top-left) where kdr frequency within An. gambiae M form larvae was particularly high

Thirty four *An. gambiae *M form females sampled as larvae in Niamey during 2003 and 53 during 2007 were also analysed. Several sites were sampled in 2003, whereas only one site was sampled in 2007, near a small stream called Gountou Yena (Figure 3). *Kdr *frequency was 32.4% in 2003 and reached 71.7% in 2007, constituting a highly significant increase (X^2 ^= 26.07, p << 0.001, df = 1). When analysing only the larval samples from the Gountou Yena stream, *kdr *frequency was 43.3% in 2003 (n = 15) and the 2003–2007 increase was still significant (X^2 ^= 8.318, p = 0.002, df = 1). In addition, considering the 2003 collections, this *kdr *frequency in the Gountou Yena area was significantly higher compared to other breeding sites in Niamey (X^2 ^= 2.957, p = 0.043, df = 1) where the mean frequency was 23.7%.

## Discussion

This study describes the first case of *kdr *mutation-harbouring *An. gambiae *populations from Niger, to contribute to the description of the spatial distribution of *kdr-w *mutation across West Africa, especially in the northern limits of *An. gambiae *distribution area where such data are very rare. Fully susceptible genotypes were found in every *An. arabiensis *sample. This result is consistent with other studies from neighbouring countries where *An. arabiensis *exhibited very few if any *kdr *alleles [21,29,32]. Despite the fact that our S form sample was too limited to describe spatial and temporal dynamics of *kdr-w *mutation, its high prevalence is nevertheless interesting. The cause of mutation maintenance without significant insecticide pressure is unknown, as the mutation frequency was actually already 39% (n = 13) before the LLIN distribution.

The collected specimens allowed a more detailed study of M form populations throughout the country. It suggests that *kdr-w *mutation was already present at a low level in M form populations before the nationwide LLIN distribution, in various localities distributed all over the sahelian zone, at longitudes ranging approximately from 1°E to 10°E and latitudes below 16°N. This finding greatly extends the area of known *kdr-*carrying M form populations, that was to date limited to more humid areas in Côte d'Ivoire [43], Ghana [36], Burkina Faso [31,32], Benin [19,50], Nigeria [22,50], Cameroon [37,50], Equatorial Guinea [41,46] and Angola [47]. The mutation frequencies encountered are consistent with studies from neighbouring countries where *kdr-w *frequency was usually low in M molecular form : 6% in Malanville, Northern Benin, near the Nigerien border [19], 0.7% in Koubri [36] and 2% in VK7 [32], southern Burkina Faso.

Based on strong supporting results, several authors [19,21,29,33,34,36,48] hypothesized that past and current agricultural use of pyrethroids and DDT for crop protection led to the selection of resistant individuals by challenging larval stages with residual insecticide products accumulating in water bodies around cultivated areas. This hypothesis was recently supported [44] by showing indirectly the presence of pesticide residues in soil and water from vegetable gardens and farms in Benin that limited the emergence rate of challenged larvae. We could relate our pre-LLIN low global *kdr *frequency to the presumed limited environmental selection pressure on *An. gambiae *populations, as the rural areas studied are usually surrounded by seasonal millet and/or sorghum subsistence cultivation for local consumption. These farming practices employ negligible amounts of pesticides. Unreliable precipitation and limited commercial demand tend to keep the use of inputs such as chemical fertilizer, pesticides and hired labour to a minimum [64]. However, several localized mosquito populations might experience more pyrethroid exposure, especially in irrigation and gardening zones where agricultural production demands and allows financially insecticide use. Urban domestic use of pyrethroids for personal protection was also suggested to favor the emergence of resistant individuals within mosquito populations [29,39]. Indeed, the highest *kdr *frequencies ever detected in M forms were found around important cities in Côte d'Ivoire [43] and Benin [19,35,44], corroborating the hypothesis of high insecticide pressure within urban environnements. In addition, it seems that M form populations breeding inside the city of Niamey also present high *kdr-w *frequencies, far greater than any analysed rural site. Some of the sampled larval populations were breeding near a small stream that crosses the city and is surrounded by year-round vegetable cultivation areas. We therefore cannot attribute potential selection effects to gardening and/or domestic insecticide use, but strongly suspect far higher insecticide exposure due to crop protection treatments. This hypothesis is sustained by the higher *kdr *frequency found near the cultivation areas compared to other larval habitats in Niamey. We conducted a basic interview in the cultivation areas, all 24 people reported repeated insecticide use along the year, bought in local market places. These multiple potential sources of pyrethroid pressure are common in many sub-Saharan urban and peri-urban areas [19,35,43,44], and are primarily due to the presence of cultivated zones in the outskirts of cities. These local situations prevent a clear identification of factors responsible for the high resistance levels detected.

Compared to the pre-LLIN 2005 collections, a significant four-fold increase of global *kdr-w *mutation frequency was observed in the 2006 wet season, around 7–8 months after the nationwide LLIN distribution, again followed by a similar four-fold increase between 2006 and 2007 wet seasons. Although suspected, the selective pressure exerted by the nationwide LLIN coverage causing the *kdr *increase within mosquito populations cannot be demonstrated (mainly due to absence of control zones). Even though it is at a quite low level, this fast and linear increase could potentially lead to high *kdr *frequencies within a few years. A similar trend was reported in *An. gambiae *S form populations from West Kenya [57] after four years of ITNs trials in a 200 km^2 ^area whereas no variation was observed in control zones without ITNs. Sharp *et al*. [58] also reported an increasing *kdr-w *frequency in M forms from Bioko Island (Equatorial Guinea) in response to an IRS programme. On the same island, Reimer *et al*. [41] found a high allelic frequency in urban and peri-urban areas one year after the beginning of residual pyrethroids spraying, that they compare to the absence of *kdr *alleles reported before the IRS programme [65]. However, it should be noted that Berzosa *et al*. [65] analysed only ten larvae at each collection site, resulting in a high probability of missing *kdr *alleles present at low to moderate frequency. Indeed, a null *kdr *frequency within 10 individuals gives a 95% Confidence Interval upper bound of 16.8%, that means a 95% probability to find no *kdr *allele in the sample although present at a frequency below 16.8% in the population. A similar rapid *kdr *frequency increase was reported in Abidjan [25,28,43] from no *kdr *alleles around 1998 (n = 30) to 39% around 1999 (n = 27) and 70% in 2004, with the last sample collected in an outdoor deltamethrin-spraying area (n = 103). Unfortunately the two first studies pre-dated the description of *An. gambiae *molecular forms so we cannot exclude an effect of variation in molecular form proportions on *kdr *frenquency. These studies and our results suggest a selection effect of large-scale insecticide-based control programmes on *kdr *mosquitoes. However, a recent study in Mali [34] showed increasing *kdr *frequencies in S forms from three villages between 1993 and 2004 that were attributed to agricultural and domestic pyrethroid use in absence of any wide-scale control programme. Also, under the hypothesis of a recent introgression of the *kdr-w *allele from S to M form in southern forest areas, we cannot rule out the possibility that the global increase we described is simply due to current spatial expansion of the mutation in West Africa.

Marked seasonality and a short rainy season condition the annual expansion of mosquito populations and the start of a new malaria transmission season. Each new malaria transmission season's increased mosquito abundance stimulates the Sahel inhabitants to use their impregnated bed nets. Indeed, the nationwide LLINs distribution campaign took place during the beginning of the dry season when usually very low mosquito densities are found throughout the sahelian zone. Our personal observations of low LLIN usage before the wet season (R. Labbo, unpublished) were confirmed by the recently published coverage and usage study [10] and are consistent with this low biting nuisance. This fact could have important implications in terms of potential selective pressure exerted by the LLIN, that would consequently be highly seasonal and co-occurrent with the fast increase of mosquito population size soon after the beginning of the rainy season. The long-term effect of such explosive patterns of mosquito populations dynamics and LLIN usage on insecticide resistance is unknown, but could potentially differ from more humid zones where populations are more stable along the year. Also, this delay between bed nets distribution and maximal usage rate (around six months after) could also have delayed the start of the resistance increase, and could explain the low kdr frequency in 2006 only a few months after the nets began to be used largely.

Concerning our finding of lower *kdr *frequency within resting compared to host-seeking mosquitoes, the most likely hypothesis is directly linked to the collection method. The resting collections used pyrethroids (0.25% tetramethrin/0.05% cyphenothrin/0.04% prallethrin) and could therefore have preferentially killed susceptible *kds/kds *females whereas at least a portion of *kdr *females could have escaped or stayed on walls and ceilings without being knocked down by the spraying. If this hypothesis was true, one should consider the collection method employed when comparing published studies or planning field collections as it could bias the *kdr *frequency values. In addition, It seems unlikely that the observed variation between collection methods is due to different sub-populations within the domestic environnement. Resting females may have taken a blood-meal one or two days before and constitute the same population that blood-seeking females collected by indoor landing catches.

Once *kdr *mutations are found in a population, one important issue would be to determine to what extent it could decrease the benefits of control programmes. The relationship between *kdr *genotypes and resistance phenotypes was partly unraveled by laboratory studies [25,28,38,45,53] and experimental huts trials [18,45], but we are far from fully understanding the role of *kdr *mutations on mosquito survival in the field. Therefore, we can only speculate on the *kdr *frequency required to measure a significant effect on insecticide resistance. Some studies give however interesting indications about the personal and/or community protection provided by insecticide materials or treatments in areas of *kdr *populations. Sharp *et al*. [58] reported a reduced infection rate after an IRS programme with pyrethroids on *An. gambiae *M form populations exhibiting around 40% *kdr *frequency, however the abundances were only decreased after a shift towards carbamate spraying. The recent study of N'guessan *et al*. [15], although conducted in semi-field conditions with experimental huts and artificially holed bed nets, gives interesting clues because the same experiments were run in two contrasting environments of *kdr *frequency in M form populations. The personal protection provided by the treated holed nets was good when *kdr *frequency was 6%, but was much decreased when kdr frequency was around 80%. In addition, in a peri-urban area of Abidjan, Côte d'Ivoire, with M form populations with a *kdr *frequency of 70% [43], a spatial spraying programme of deltamethrin and fenitrothion in conjunction with deltamethrin-treated bed net usage in a french military camp did not significantly reduce the mosquito biting rates.

Therefore, as the present results indicate still low *kdr *frequencies mainly in the heterozygous state, it is unlikely that the current spread of the mutation has to date any significant effect on resistance phenotype and resulting efficacy of LLIN. However, it is feared that this continuous spread of *kdr-w *mutation could rapidly impede current efforts to reduce malaria transmission by implementing large-scale pyrethroid-treated nets coverage.

## Conclusion

These results are of prime importance in our effort to document multiple effects of operational control programmes on mosquito vectors, and to conceive sustainable control strategies for the future. As an increasing number of African countries plan to develop and scale up malaria control strategies including large vector control implementation, continued monitoring for insecticide resistance will be of utmost importance in a context of increasing extensive pyrethroid exposure. The documentation of the factors contributing to resistance selection within those populations is also highly important. Nevertheless, the long-term sustainability of such programmes will not be achieved without the availability of alternative insecticidal compounds that could replace pyrethroids for bed net impregnation where they would appear inefficient due to *kdr *mutations and/or other resistance mechanisms.

## Competing interests

The authors declare that they have no competing interests.

## Authors' contributions

CC carried out the molecular processing, the analysis and interpretation of data, and contributed to the drafting of the manuscript; RL participated to the conception of the study and the field samples collection and identification; IA participated to the collection and identification of the samples and the molecular processing; JBD conceived and coordinated the study, and contributed to the interpretation of data and the drafting of the manuscript. All authors read and approved the final manuscript.
